# Supplementary material for: Magnetically Actuated Microscaffold with Controllable Magnetization and Morphology for Regeneration of Osteochondral Tissue
Source: Micromachines (Basel). 2023 Feb 11;14(2):434. doi: 10.3390/mi14020434 (PMC9959313; doi:10.3390/mi14020434)
Supplement: Supplementary file 1 [file micromachines-14-00434-s001.zip › micromachines-2181037-supplementary material_figure S.pdf]

micromachines

MDPI

Supplementary material

Magnetically actuated microscaffold with controllable magnetization and morphology for regeneration of osteochondral tissue

Junhyeok Lee [1,2], Hyeong-Woo Song [3], Kim Tien Nguyen [3], Seokjae Kim [1,3], Minghui Nan [2,3], Jong-Oh Park [1,3], Gwangjun Go [3]* and Eunpyo Choi [1,2,3]*

[1] School of Mechanical Engineering, Chonnam National University, 77 Yongbong-ro, Buk-gu, Gwangju 61186, South Korea
[2] Robot Research Initiative, Chonnam National University, 77 Yongbong-ro, Buk-gu, Gwangju 61186, Republic of Korea
[3] Korea Institute of Medical Microrobotics, 43-26, Cheomdangwagi-ro 208-beon-gil, Buk-gu, Gwangju 61011, South Korea
* Correspondence: gwangjun124@kimiro.re.kr (G. G.); eunpyochoi@jnu.ac.kr (E.C);

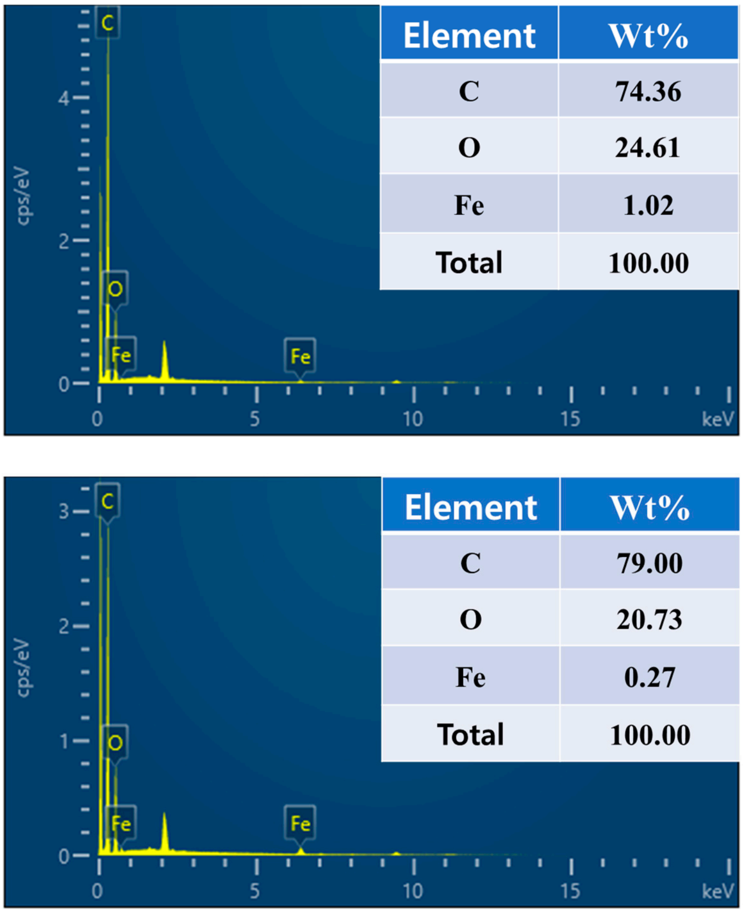

Figure S1. Graphs and table of element signals for MAM-CR (top) and MAM-SBR (bottom)

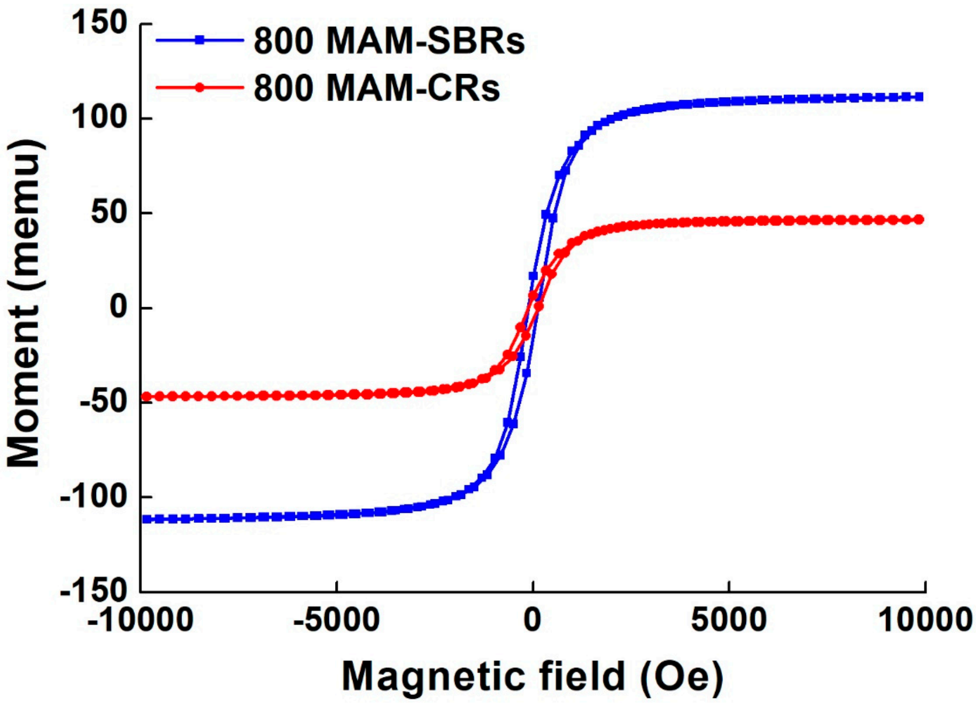

Figure S2. Magnetization curves of 800 MAM-CRs and 800 MAM-SBRs

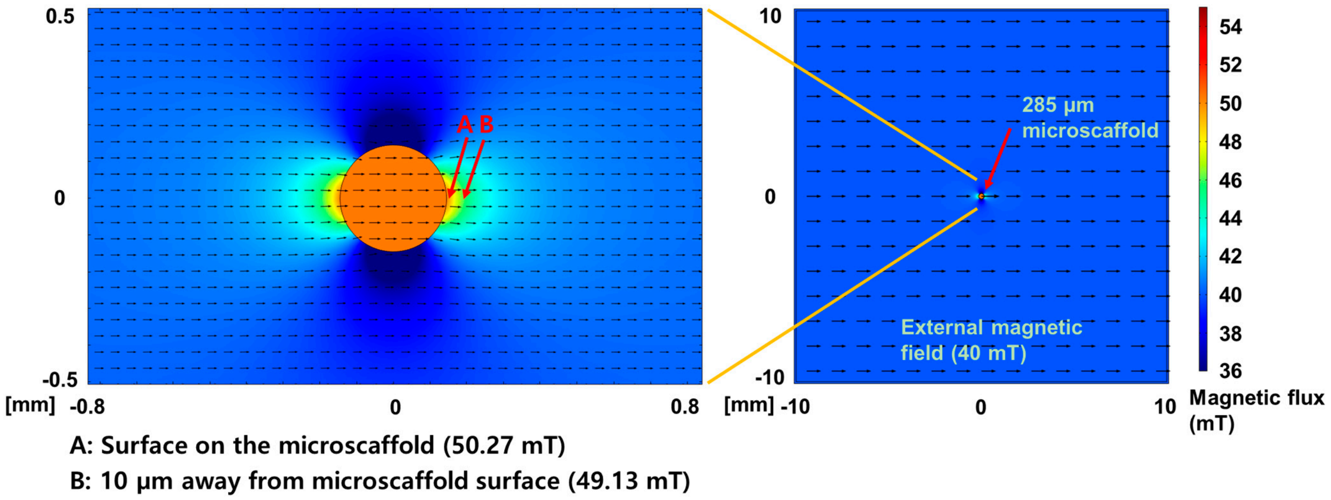

Figure S3. Numerical simulation showing magnetic fields map of the magnetized microscaffold under external magnetic fields. Black arrows indicate direction of magnetic fields

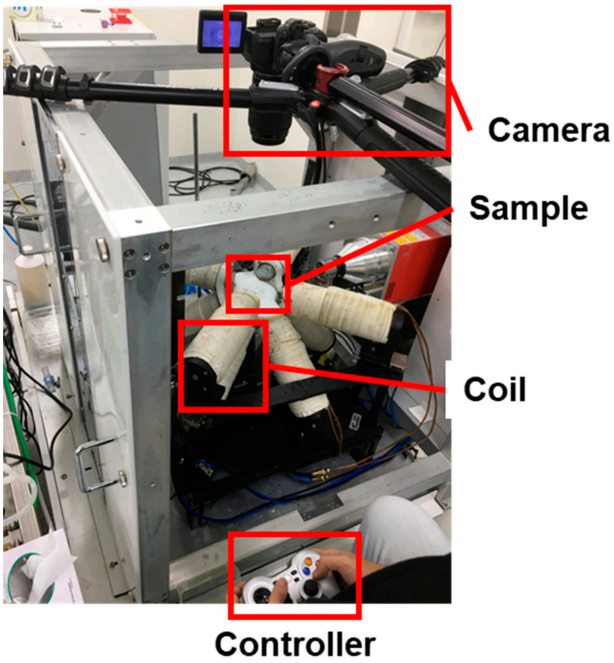

Figure S4. Experimental setup for magnetic actuation of the microscaffolds

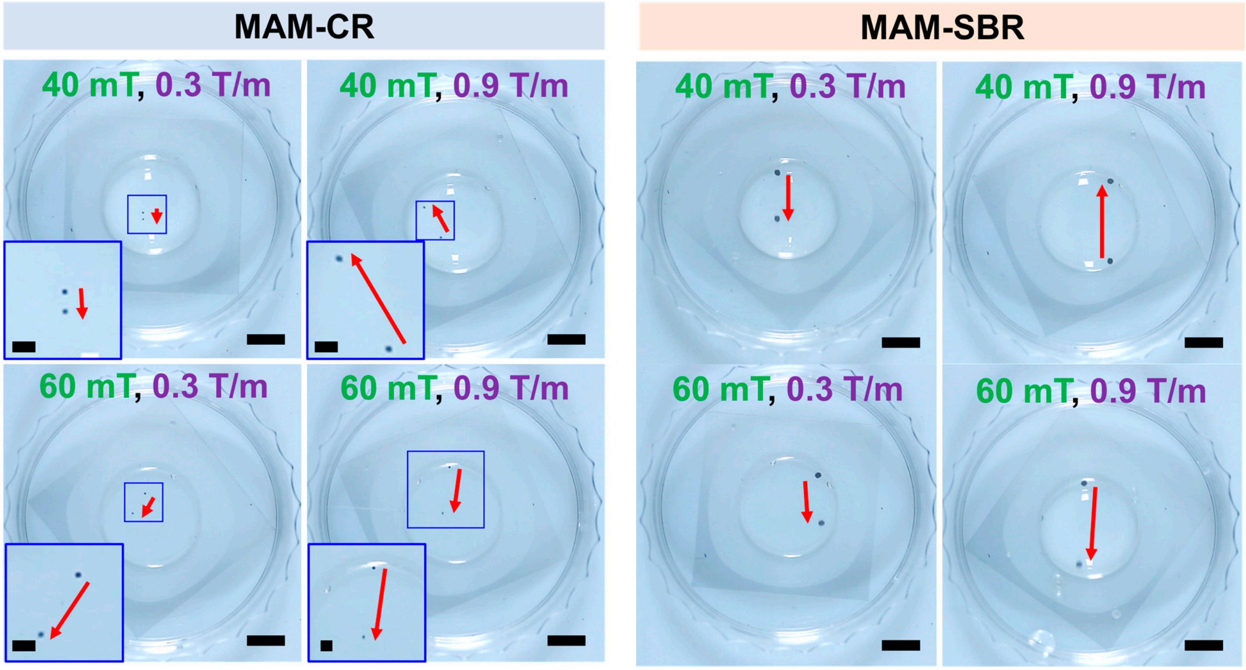

Figure S5. Timelapse images for mobility of MAM-CR and MAM-SBR at different magnetic fields (40 and 60 mT) and gradients (0.3 and 0.9 T/m). Red arrows indicate moving distances of the microscaffolds for one second. Scale bars are 5 mm. The blue boxes in MAM-CR are shown as insets. Scale bar of the inset images is 1 mm.
